# A Culturally Tailored Physical Activity Intervention for Hispanic Adults Living With Type 2 Diabetes: Pre-Post Pilot Feasibility Study

**DOI:** 10.2196/62876

**Published:** 2025-06-10

**Authors:** Julio Loya, David O Garcia, Adriana Maldonado, Edgar Villavicencio

**Affiliations:** 1 Advanced Nursing Practice and Science, College of Nursing, University of Arizona, 1305 North Martin Avenue, Tucson, AZ, 85721-0203, United States, 1 520-621-9112; 2 Health Promotion Sciences, Mel & Enid Zuckerman College of Public Health, University of Arizona, Tucson, AZ, United States

**Keywords:** type 2 diabetes, physical activity, Hispanic adults, intervention, diabetes mellitus, metabolic disease, United States, lifestyle modification, pilot intervention, community-engaged, self-efficacy

## Abstract

**Background:**

Type 2 diabetes mellitus (T2DM) is a metabolic disease that affects over 38 million adults in the United States, who are disproportionately Hispanic.

**Objective:**

This study describes the development and implementation of Salud Paso por Paso, a culturally tailored and linguistically appropriate intervention to increase engagement in physical activity (PA) for Hispanic adults living with T2DM.

**Methods:**

Participants were enrolled in a 6-week pre-post pilot test of a culturally tailored intervention that included sessions covering different aspects of PA and T2DM. Participants were recruited at a local free clinic. Nonparametric paired-sample Wilcoxon signed-rank tests were used to examine differences between pre- and postintervention measures.

**Results:**

Twenty-one participants were recruited, and 19 (90.5%) completed the intervention. Participants significantly increased average hours spent in moderate PA, by 3.16 hours (from 4.73, SD 3.79 minutes to 9.63, SD 6.39 minutes; *Z*=−3.52; *P*<.001), average steps per week (from 23,006.38, SD 14,357.13 steps to 43,000.81, SD 30,237.17 steps; *Z*=−2.79; *P*=.005), and minutes per week of PA (from 105.94, SD 72.23 minutes to 224.19, SD 167.85 minutes; *Z*=−3.36; *P*<.001).

**Conclusions:**

Developing effective culturally tailored interventions that can ameliorate the deleterious effects of T2DM in Hispanic adults is an important strategy to promote health equity. The Salud Paso por Paso intervention is an effective way to promote PA in Hispanic adults living with T2DM.

## Introduction

Diabetes mellitus is a metabolic disease that affects approximately 38 million individuals in the United States [1]. Among individuals living with diabetes, over 90% are classified as having type 2 diabetes mellitus (T2DM) [1]. Risk factors for T2DM include age (45 years or older), having immediate family members with T2DM, having prediabetes, having a history of gestational diabetes, being of Hispanic/Latino descent, having overweight or obesity, and having a low level of physical activity (PA) [2]. Estimates show that T2DM prevalence in Hispanic individuals is approximately 10.3% (compared to 8.5% for non-Hispanic White individuals) [3]. Hispanic adults are disproportionally affected by T2DM and exhibit disparities in diabetes-related complications, including nephropathy, retinopathy, and amputations [4].

Lifestyle modification, including changes in PA, is one of the cornerstones to enhance health outcomes for people living with T2DM. Current PA guidelines state that for substantial health benefits, adults should engage in at least 150 minutes to 300 minutes a week of moderate-intensity aerobic PA, or 75 minutes to 150 minutes a week of vigorous-intensity aerobic PA [5]. Regular PA can reduce mortality risk among adults with T2DM by improving cardiovascular fitness, enhancing glycemic control, improving vascular function, and contributing to weight management [6,7]. Despite the benefits of regular PA, almost 32% of US adults with diagnosed T2DM are physically inactive, defined as engaging in less than ten minutes a week of moderate or vigorous activity [3]. Within ethnic minority populations, estimates show Hispanic adults having the highest prevalence of physical inactivity at 32.1% [8]. Evidence suggests that there is a wide variation in cultural tailoring of interventions, with strategies ranging from adapting previously evaluated interventions in Spanish, such as the Diabetes Prevention Program, to using a formalized community-engaged research approach to develop intervention content [9]. Despite recognizing that culturally tailoring interventions for implementation with Hispanic adults with T2DM may be effective, the heterogeneity of approaches to culturally tailoring interventions shows the need for more rigorous approaches [9]. Effective strategies are needed to promote moderate-intensity PA among Hispanic adults living with T2DM [10].

There is evidence suggesting intervention programs to prevent or delay T2DM onset are not readily accessible to ethnic and racial minorities, which may contribute to diabetes disparities [9]. Efforts to reach high-risk populations such as Hispanic adults with T2DM include the development of culturally tailored or adapted interventions [9]. Culturally tailored interventions involve more than linguistic translation of intervention components. Rather, cultural tailoring of interventions should include domains such as attention to family orientation and fostering a strong relationship with the health care provider, along with empowerment, a sense of control, and respect for religion and folk beliefs [11]. Additionally, to culturally tailor interventions, it is important to use principles of community engagement, as these are well suited to engage stakeholders from the population of interest [11]. Culturally tailoring intervention components such as literacy modifications and delivering interventions in appropriate community settings may be effective in modifying behavior in Hispanic populations [9]. Collectively, these data underscore the need for tailored and linguistically appropriate interventions among Hispanic adults living with T2DM [9]. Thus, the purpose of this study is to describe the development and implementation of Salud Paso por Paso (Health Step by Step), a culturally tailored and linguistically appropriate intervention designed according to principles of community engagement to enhance self-efficacy and increase engagement in PA among Hispanic adults living with T2DM.

## Methods

### Study Design, Sample Size, and Participant Recruitment

A six-week pre-post pilot test study was designed to examine the feasibility and preliminary acceptability of Salud Paso por Paso. Inclusion criteria included a confirmed diagnosis of T2DM by a health care provider, the ability to speak English or Spanish, and an ongoing sedentary lifestyle (ie, lying or sitting down with little to no exercise in the past 6 months) [12]. The recruitment period was from October 2019 through February 2020. The initial recruitment goal was to enroll 24 participants to account for attrition. The primary objective of this study was to assess the feasibility of the intervention. As such, no formal sample size calculations were made. However, there is evidence to suggest that a minimum of 12 subjects per group can be considered for pilot studies [13]. Participants were recruited through flyers and generalized announcements made at the recruitment site. Recruitment only occurred at the clinic. One of the particular characteristics of the clinic is that it is only open 2 days a week, in the afternoon. Additionally, the clinic closes for the week during holidays. While the recruitment time period spanned October 2019 to February 2020, there were 3 weeks when the clinic was not open due to holidays. Furthermore, due to holiday travel, there were decreased numbers of attendees of clinic sessions when the clinic was open.

### Ethical Considerations

Written informed consent was obtained from participants prior to study participation. Participants provided consent in the language of their choice (English or Spanish) using written consent documents. Participants had the opportunity to ask questions and obtain clarification regarding study components and study purpose. Participants received compensation in the form of gift cards (US $80) for their participation in the study. Data included in this study are deidentified to protect participant privacy and confidentiality. Approval for this study was obtained from the University of Missouri Institutional Review Board (2013903).

### Study Setting

The intervention was delivered at a free community clinic located within a neighborhood center complex in Tucson, Arizona. The neighborhood center complex is located next to a public bus transit center. Key stakeholders identified the location as a facilitator for participants to access clinic services. The clinic provides health care services to community members and is staffed by volunteer health care providers, including physicians, physician assistants, nurse practitioners, registered nurses, and certified diabetes educators. Additionally, PA activities were completed by using the current infrastructure of the neighborhood complex; this complex has ample space with sidewalks to facilitate walking. Participants were asked to walk around the neighborhood complex in the designated exercise areas and walking paths.

### Intervention Development

There were several efforts to culturally tailor the intervention component for this study. Input for development of the Salud Paso por Paso intervention was provided by community members and staff from the community-based clinic (ie, medical staff, nurse volunteers, and a diabetes nurse educator). Key stakeholders were identified through their roles at the clinic (eg, experienced care providers, long-term volunteers, clinic leaders, and long-term patients). Four informal meetings were held with key stakeholders from the clinic to solicit input for intervention development. Based on stakeholder feedback, the intervention was tailored to the participants’ Hispanic background. Stakeholders identified prevalent participant characteristics that included Spanish as the primary language and lower levels of literacy. All study materials were modified for delivery in Spanish and the educational materials were reviewed and adapted to a fifth grade reading level. To increase ease of participant attendance at educational and PA sessions, the educational component of the intervention was delivered in the waiting room of the clinic, and the PA component of the intervention was performed around the neighborhood center complex.

Intervention development was guided by social cognitive theory [14,15] to enhance self-efficacy for increasing PA in accordance with established guidelines from the American Diabetes Association [16] and American Heart Association [17]. The PA component of the intervention was purposefully modified for participants, with a goal of increasing walking incrementally to 150 minutes per week to reduce risk of injury. Table 1 provides an overview of the Salud Paso por Paso sessions and PA goals.

**Table 1.** Intervention activities for physical activity (PA).

Session	Duration	Components	Activities
1. Changing PA behavior	60 min	Welcome to the Salud Paso por Paso study Discussion on motivation for participants to engage in PA Intervention orientation Materials provided to participants (eg pedometers, paper logs) PA recommendations based on American Heart Association guidelines Participant PA instructions for following week	Demonstration of warm-up process for safely engaging in PA by principal investigator and participants (eg 5 minutes of walking at a casual pace) Participants instructed to engage in at least 5 minutes of walking at a casual pace for 5 days during this week
2. Potential barriers to engaging in PA	45 mins	Group discussion of potential barriers to PA Group discussion on solutions to overcome potential barriers to PA Group discussion on ability to engage and maintain PA during last week Reviewed PA logs	5 minutes of walking at a casual pace Demonstration of moderate-intensity PA by principal investigator and participants for 5 minutes (eg brisk walking—at least 4 km per hour) Participants were instructed to engage in 5 minutes of walking at a casual pace and 5 minutes of moderate-intensity walking for 5 days during this week
3. Motivation for PA	45 mins	Group discussion of participant motivators for PA Group discussion on strategies to remain motivated for PA Group discussion on ability to engage and maintain PA during last week Reviewed PA logs	5 minutes of walking at a casual pace by principal investigator and participants 10 minutes of moderate-intensity PA by principal investigator and participants Participants were instructed to engage in 5 minutes of walking at a casual pace and 10 minutes of moderate-intensity walking for 5 days during this week
4. Potential setbacks	45 mins	Group discussion on potential setbacks that occur during PA behavior change Group discussion on how to deal with setbacks during PA behavior change Group discussion on ability to engage and maintain PA during last week Reviewed PA logs	5 minutes of walking at a casual pace by principal investigator and participants 15 minutes of moderate-intensity PA by principal investigator and participants Participants were instructed to engage in 5 minutes of walking at a casual pace and 15 minutes of moderate-intensity walking for 5 days during this week
5. Family/social support	45 mins	Group discussion on potential sources of support during PA behavior change Group discussion on capitalizing on support from family and social networks during PA behavior change Group discussion on strategies to engage family and friends in PA Group discussion on ability to engage and maintain PA during last week Reviewed PA logs	5 minutes of walking at a casual pace by principal investigator and participants 20 minutes of moderate-intensity PA by principal investigator and participants Participants were instructed to engage in 5 minutes of walking at a casual pace and 20 minutes of moderate-intensity walking for 5 days during this week
6. A better future	60 mins	Group discussion on achievements and successes from participation in Salud Paso por Paso Group discussion on long-term sustainment of PA change Group discussion on ability to engage and maintain PA during last week Reviewed PA logs	5 minutes of walking at a casual pace by principal investigator and participants 25 minutes of moderate-intensity PA by principal investigator and participants Participants were instructed to engage in 5 minutes of walking at a casual pace and 25 minutes of moderate-intensity walking for 5 days during this week

### Measures

PA outcomes were assessed by (1) 7-day PA recall at baseline and after the intervention, (2) self-efficacy for PA at baseline and after the intervention, (3) total number of steps per week, and (4) total minutes of PA per week per participant. Demographic and health characteristics, including acculturation scores, were collected at baseline to describe the study sample.

#### Weekly Exercise Logs and PA Recall

Frequency, number of steps, and number of minutes spent in PA each week were recorded by participants using paper and pencil logs. Participants received a pedometer and instructions on how and when to wear the pedometer. The pedometer model was an Omron Alvita USB Pedometer (model HJ-322U). Participants were asked to record steps per day with the pedometer and minutes spent in PA and to bring the logs to each weekly session. The principal investigator administered the 7-Day Physical Activity Recall (7-Day PAR) paper questionnaire during the baseline and postintervention assessments. The 7-Day PAR measures time spent in PA, strength, and flexibility activities for the 7 days prior to questionnaire completion [18]. The 7-Day PAR has been validated for use with adults and has been translated into Spanish [19,20].

#### Self-Efficacy

Participants completed the Self-Efficacy for Exercise Behaviors Scale (SEEBS) at baseline and after the intervention. The SEEBS scores were used to measure exercise self-efficacy. The SEEBS questions are divided by subscales for “making time for exercise” and “sticking to it.” Self-efficacy for participants was defined as the perception by the participant that they could engage in exercise behaviors. The SEEBS measures self-efficacy on a Likert scale from 1 to 4, with higher scores relating to higher self-efficacy, and it has been validated for use in Hispanic populations [21]. Lower SEEBS scores signify that participants believe they cannot accomplish the behaviors; conversely, higher SEEBS scores signify participants’ belief that they can accomplish the behaviors.

#### Acculturation

Participants completed the Short Acculturation Scale for Hispanics (SASH). The SASH measures changes that Hispanic individuals experience in values, norms, attitudes, and behaviors when exposed to mainstream cultural patterns of the United States [22]. The scale measures acculturation on a 5-point Likert scale, with a value of 1 corresponding to “very Hispanic” and a value of 5 corresponding to “very American.”

#### Demographic Data and Health Information

Age, sex, ethnicity, birthplace, marital status, educational level, income level, and additional health conditions were obtained through self-reporting.

#### Feasibility

Feasibility was evaluated based on the ability to recruit participants, attendance at each weekly session, and completion of intervention components by participants, which included daily logs documenting PA. A meta-analysis found mean retention rates of 75% (SD 13%) for intervention groups and mean adherence rates of 61% (SD 21%) for PA studies [23]. For this study, retention and adherence goals were set at 80% due to the characteristics of the target sample. Potential participants for this study were people who spend time living and traveling between Mexico and Tucson, Arizona. Evidence suggests that current national- and state-level surveys in the United States and Mexico only capture information regarding stationary Mexican immigrant and returned migrant populations [24]. Some of the potential participants cited their inability to predict when they would be present in the United States as the main barrier to participating in this study. Due to the mobility of this sample of the population, the principal investigator determined the retention and adherence rates for the small sample size. The retention goal was for 20 participants to complete the intervention. The goal for participant intervention adherence, or attendance to weekly sessions and completion of PA logs, was to attend 5 of 6 (>80%) weekly sessions and complete 5 of 6 weekly PA logs.

### Data Collection

The principal investigator shared all aspects of the study with potential participants by meeting individually with them in a private area of the clinic. All data were collected using paper and pencil surveys. All participants received a health screening and physical examination, and they were cleared for study participation by a physician or nurse practitioner prior to participating in intervention and PA sessions. The first wave of intervention delivery occurred from the first week of March 2020 through the second week of April 2020. The second wave of intervention delivery occurred from the third week of April 2020 through the fourth week of May 2020. Notably, this is the time when the COVID-19 pandemic restrictions began in earnest in Arizona; these restrictions included a requirement for social distancing and masking and avoiding unnecessary exposure to other people in public.

### Statistical Analysis

Data were analyzed using SPSS Statistics (version 29; IBM Corp) and screened for normality, missing data, and outliers. Ranges, means, and SDs for demographic data, self-efficacy scores, and PA frequency were calculated at baseline and at intervention completion. Nonparametric paired-sample Wilcoxon signed-rank tests were performed to examine differences between pre- and postintervention measures on 7-Day PAR results, PA self-efficacy scores, self-reported steps per week, and self-reported minutes of PA per week.

## Results

### Characteristics of Study Participants

Twenty-four potential participants were screened between mid-October 2019 and February 2020. After recruitment was completed, 3 participants were unable to begin the study as they had to unexpectedly travel to Mexico for various reasons, including to provide care for family members. A total of 21 (88%) participants ultimately enrolled in the Salud Paso por Paso study. Participant characteristics are described in Table 2. These characteristics describe all participants who originally enrolled, as they provided demographic information at the beginning of the study. Briefly, participants ranged in age from 30 to 75 years (mean 53, SD 11.8 years). The majority (n=19, 90%) of participants were female, and most (n=16, 76%) were married. All participants except one identified Mexico as their country of origin. Most participants had an income level below US $35,000, with 9 participants reporting an income level less than US $15,000. Over half the participants had completed high school or had less than a high school education. Participants had lived in the United States from 3 months to 35 years (mean 14.9, SD 11.25 years). Over half the participants reported another chronic health condition in addition to T2DM. Participants had a mean SASH score of 1.53 (SD 0.744) at baseline, indicating a low level of acculturation.

**Table 2.** Salud Paso por Paso participant demographic and health characteristics (n=21).

Characteristics		Values
Age (years), mean (SD)		53.0 (11.8)
Gender, n (%)		
	Female	19 (90)
	Male	2 (10)
Ethnicity, n (%)		
	Mexican	20 (95)
	Dominican	1 (5)
Marital status, n (%)		
	Single/divorced	4 (19)
	Married/widowed	16 (76)
	No answer	1 (5)
Yearly household income, n (%)		
	<US $14,999	9 (43)
	>US $15,000-$34,999	4 (19)
	>US $35,000	2 (10)
	No answer	6 (29)
Education level, n (%)		
	Less than high school	7 (33)
	High school	7 (33)
	College	6 (28)
	No answer	1 (5)
Years living in the United States, mean (SD)		14.9 (11.3)
Primary language at home, n (%)		
	Spanish	21 (100)
Other chronic health conditions, n (%)		
	Present (prediabetes, hypertension, other)	12 (57)
	Not present or did not answer	9 (43)
Short Acculturation Scale for Hispanics score, mean (SD)		1.53 (0.7)

### Feasibility

Twenty-four participants were recruited over a period of 14 weeks, with 21 participants completing the initial intervention measures. Nineteen of 21 (90%) participants who enrolled in the study attended all weekly face-to-face intervention sessions. One participant attended the first week of the intervention but withdrew from the study due to work schedule conflicts. Another participant withdrew from the study in week 3 due to work schedule conflicts as well as the need to take care of a family member.

### 7-Day PAR Results

Participants significantly increased the average hours spent in moderate PA, by 3.16 hours (from 4.73, SD 3.79 to 9.63, SD 6.39 hours; *Z*=−3.52, *P*<.001). There were no significant results for vigorous PA or very vigorous PA (Table 3).

**Table 3.** 7-Day Physical Activity Recall (PAR) results (n=16; not all participants were included due to the inability of some to recall end point activity).

		Hours, mean (SD; 95% CI)	*Z* score	*P* value
7-Day PAR moderate			–3.52	<.001
	Baseline	4.73 (3.79; 2.71 to 6.76)		
	End point	9.63 (6.39; 6.22 to 13.03)		
7-Day PAR vigorous			–0.54	.59
	Baseline	1.75 (4.27; –0.52 to 4.02)		
	End point	1.17 (3.52; –0.70 to 3.05)		
7-Day PAR very vigorous			–1.0	.32
	Baseline	0 (0; 0 to 0)		
	End point	0.06 (0.25; –0.07 to 0.20)		

### SEEBS Results

The preintervention SEEBS mean score for participants was 3.53 (SD 0.65), and the postintervention mean score was 3.6 (SD 0.42). Subscale scores before and after the intervention were similar for “making time for exercise” and “sticking to it” (Table 4). The mean score >3.5 suggests that all the participants had high self-efficacy to engage in exercise prior to starting the intervention. Overall, there were no significant differences before and after the intervention in self-efficacy scores (*Z*=−0.764; *P*=.44).

**Table 4.** Self-Efficacy for Exercise Behaviors Scale (SEEBS) results.

		Score, mean (SD; 95% CI)	*Z* score	*P* value
All SEEBS factors			–0.76	.45
	Baseline	3.53 (0.65; 3.22‐3.84)		
	Postintervention	3.60 (0.42; 3.40‐3.80)		
Making time for exercise			–0.231	.82
	Baseline	3.65 (0.55; 3.38‐3.91)		
	Postintervention	3.65 (0.39; 3.46‐3.84)		
Stick to it			–1.026	.31
	Baseline	3.45 (0.74; 3.09‐3.80)		
	Postintervention	3.57 (0.46; 3.35‐3.79)		

### PA Steps and Minutes per Week

The participants significantly increased their average number of steps per weeks from 23,006.38 (SD 14,357.13) to 43,000.81 (SD 30,237.17; *Z*=−2.79; *P*=.005). Similarly, participants reported significant increases in the amount of time they engaged in PA per week, from 105.94 (SD 72.23) minutes at baseline to 224.19 (SD 167.85) minutes at week 6 (*Z*=−3.36, *P*<.001; Table 5).

**Table 5.** Steps and minutes of physical activity per week.

		Values, mean (SD; 95% CI)	*Z* score	*P* value
Steps			–2.79	.005
	Baseline	23,006.38 (14,357.13; 15,356‐30,656.75)		
	End point	43,000.81 (30,237.17; 26,888.56‐59,113.06)		
Minutes of physical activity			–3.36	<.001
	Baseline	105.94 (72.23; 67.45‐144.42)		
	End point	224.19 (167.85; 134.75‐313.63)		

## Discussion

### Principal Findings

The purpose of this pilot study was to examine the feasibility and preliminary effectiveness of a culturally tailored PA intervention, Salud Paso por Paso*,* in a group of Hispanic adults living with T2DM. Findings indicate adherence to the PA intervention was satisfactory in the priority population and participants showed a significant increase in steps and minutes of PA per week from before to after the intervention.

Participants in this study had low acculturation levels, reflecting the participants’ strong identification with their Hispanic heritage. Low levels of acculturation have been associated with lower levels of self-rated health and lower use of medical services in Hispanic people [25]. However, participants in this study, despite lower levels of acculturation, successfully increased their PA from before to after the intervention. Participants in this study had high self-efficacy for exercise at baseline. Previous research indicates that higher self-efficacy for exercise is associated with increased PA [26]. In this context, participants may have had the desire and ability to engage in PA, but may not have had the opportunity to do so prior to enrolling in this study. Participants may have benefited from the structure and accountability provided through their participation in this study. It is notable that 19 participants (90%) completed the intervention, even considering the negative impact that the COVID-19 pandemic has had not only on conducting research, but on PA levels [27].

While participants showed increased engagement in PA, evidence suggests that the COVID-19 pandemic had a negative effect on PA, with a declining trend in PA engagement across participants from different countries [28]. This decline in PA engagement was also reflected in the United States, where Hispanic adults engaged in less PA when compared to White adults [29]. Participants in this study expressed interest in continuing with the intervention behaviors after the intervention ended. Some of the participants stated that they would be willing to enroll in a different study with a longer duration, as they perceived the 6-week intervention as ending quickly.

Modest increases in PA by participants helped improve adherence to PA recommendations at the completion of the study. The intervention was unique because it was delivered in the clinic and a neighborhood center complex, a location that was accessible and familiar for participants as this is where they typically seek health care services. Strengths of this research included its community-partnered approach and the researcher sharing the cultural and linguistic background of the participants. While the findings of this study are promising regarding increasing PA, there were limitations. This was a small, noncontrolled pilot study. A prospective randomized study with a longer duration is needed to further examine intervention efficacy. In addition, refinement of study materials as well as determining whether PA was maintained would strengthen the findings of a future iteration of this study. Another limitation is the potential negative impact the COVID-19 pandemic had on intervention fidelity. The intervention study began just 2 weeks before an executive order from the governor of Arizona recommended social distancing and mask precautions [30]. While PA was an approved activity under this order and all appropriate precautions (eg physical distancing) were maintained, many of the participants expressed concern about engaging in PA due to the increased risk of contracting COVID-19.

### Conclusions

The need for effective interventions that promote PA in Hispanic adults, and the general population of individuals living with T2DM, is considerable. As the Salud Paso por Paso intervention was being conducted, many participants expressed interest in learning more about other behaviors that can enhance T2DM outcomes, such as nutrition. Significant results for PA outcomes, specifically 7-Day PAR increases in moderate PA and increases in number of steps and PA minutes, support a prospective randomized controlled study to examine the efficacy of Salud Paso por Paso among Hispanic adults living with T2DM. Future research should include measures to examine the ability for long-term sustainment of PA improvements. Since this was a feasibility study, results from this study could inform the planning and completion of a randomized controlled trial for future research. Developing effective interventions that can ameliorate the deleterious effects of T2DM in Hispanic adults is an important strategy to promote health equity.

## Abbreviations

7-Day PAR: 7 Day Physical Activity Recall
PA: physical activity
SASH: Short Acculturation Scale for Hispanics
SEEBS: Self-Efficacy for Exercise Behaviors Scale
T2DM: type 2 diabetes mellitus
